# “HLA-C: evolution, epigenetics, and pathological implications in the major histocompatibility complex”

**DOI:** 10.3389/fgene.2023.1206034

**Published:** 2023-07-03

**Authors:** Erick Velastegui, Edwin Vera, Wim Vanden Berghe, Mindy S. Muñoz, Andrea Orellana-Manzano

**Affiliations:** ^1^ Escuela Politécnica Nacional, Departamento de Ciencias de los Alimentos y Biotecnología, Facultad de Ingeniería Química y Agroindustria, Quito, Ecuador; ^2^ Epigenetic Signaling Lab, Faculty Biomedical Sciences, PPES, University of Antwerp, Antwerp, Belgium; ^3^ Facultad de Medicina Clínica Alemana, Universidad del Desarrollo, Santiago, Chile; ^4^ Escuela Superior Politécnica del Litoral, Laboratorio para investigaciones biomédicas, Facultad de Ciencias de la Vida (FCV), Guayaquil, Ecuador

**Keywords:** HLA-C, major histocompatibility complex (MCH), immune response, epigenetic modifications, KIR

## Abstract

HLA-C, a gene located within the major histocompatibility complex, has emerged as a prominent target in biomedical research due to its involvement in various diseases, including cancer and autoimmune disorders; even though its recent addition to the MHC, the interaction between HLA-C and KIR is crucial for immune responses, particularly in viral infections. This review provides an overview of the structure, origin, function, and pathological implications of HLA-C in the major histocompatibility complex. In the last decade, we systematically reviewed original publications from Pubmed, ScienceDirect, Scopus, and Google Scholar. Our findings reveal that genetic variations in HLA-C can determine susceptibility or resistance to certain diseases. However, the first four exons of HLA-C are particularly susceptible to epigenetic modifications, which can lead to gene silencing and alterations in immune function. These alterations can manifest in diseases such as alopecia areata and psoriasis and can also impact susceptibility to cancer and the effectiveness of cancer treatments. By comprehending the intricate interplay between genetic and epigenetic factors that regulate HLA-C expression, researchers may develop novel strategies for preventing and treating diseases associated with HLA-C dysregulation.

## 1 Introduction

The human leukocyte antigen complex (HLA) or also called the human major histocompatibility complex (MHC), is a genetic region present in humans (Mallia et al., 2012). *Homo sapiens* comprises a broad set of more than 200 genes located in a highly polymorphic region of the short arm (p) of chromosome 6. HLA is involved in the body’s immune response. About 40 of its genes are specifically encoded in leukocyte antigens, and the rest participate in their functionality. Interestingly, several genes in this complex have no known function in the immune system. The complex is divided into classes I, II and III, which participate in the immune response in structure and function. More specifically, there are around 20 genes within class I, but the main ones are called class 1a, such as HLA-A, HLA-B, and HLA-C. These three are fundamental agents in the mechanism of innate immune response when interacting with NK (Natural killers) with their killer-cell immunoglobulin-like (KIR) receptors, and with T CD8^+^ and indirectly with cells T CD4^+^, as part of the adaptive immune response (Klein and Sato, 2000; Aguiar et al., 2019).

**FIGURE 1:**
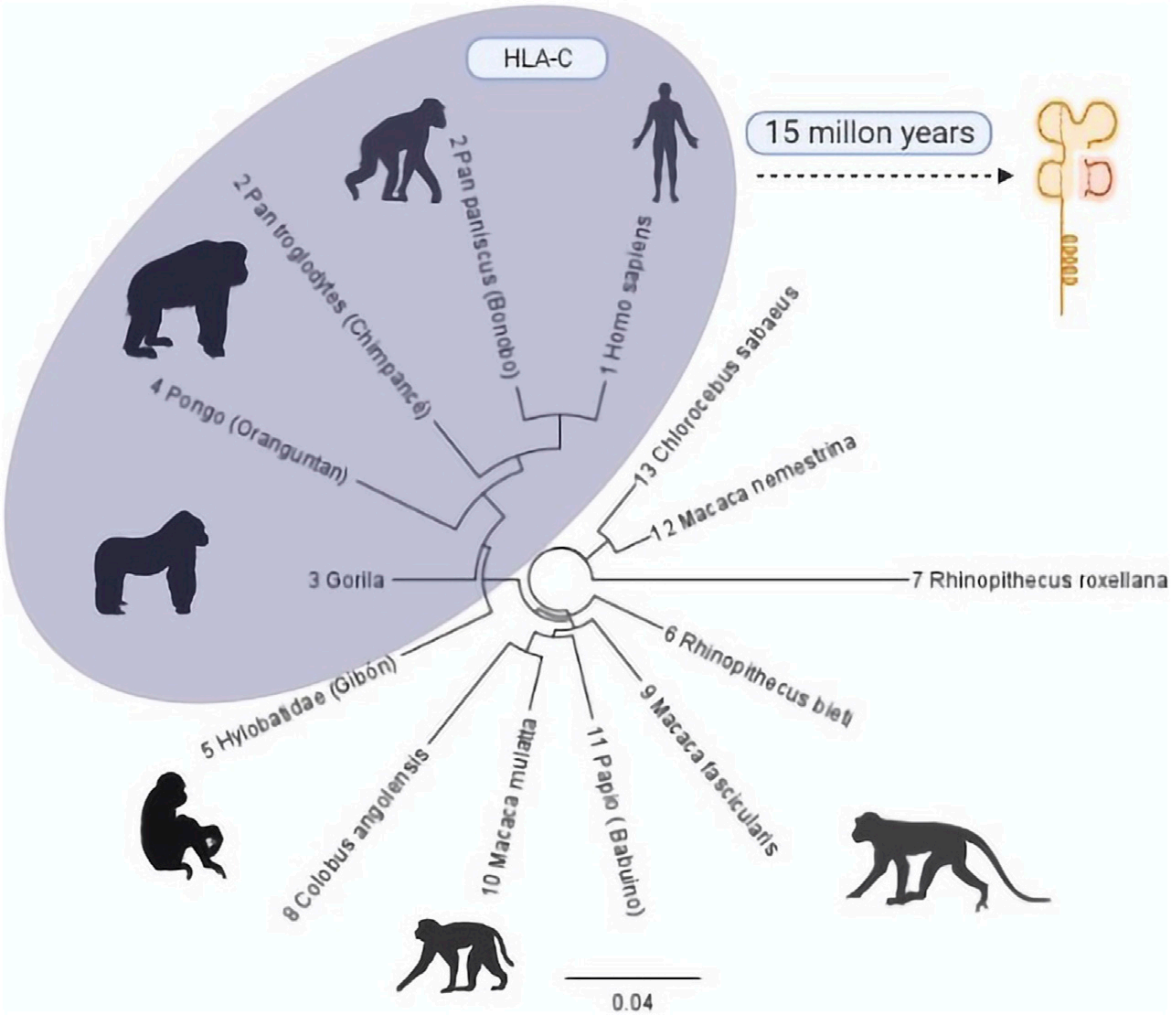
Phylogenetic tree of the evolutionary process of HLA-C in primates. Primates with some HLA-C allotypes are grouped in the grey region. The number before each species describes the evolutionary closeness of each one with *Homo sapiens* (Mallia et al., 2012) compared to the similarity of regions within the class I type C histocompatibility complex. Phylogenic Tree: Geneious Prime 20221.1 [https://www.geneious.com]. Illustrations Created with BioRender.com.

On an evolutionary scale, HLA-C emerged 10 or 15 million years ago in an ancestor shared with African apes, humans, and orangutans that until now have only one allotype, C1. This is preserved in great apes such as gorillas, chimpanzees, bonobos, and humans but with an additional allotype (HLA-C2) (Adams et al., 1999; Li et al., 2021). It is estimated that it was generated by the duplication of HLA-B because, although both are highly polymorphic, they have distinct preserved regions (Heijmans et al., 2020; Li et al., 2021). The similarity between these two genes is so remarkable that the proximal promoter regions of HLA-B and HLA-C differ by only one base pair (G/A) (Figure 1).

Simultaneously to developing HLA-C in higher primates, KIR receptors by the immune response also evolved, based on sequence information from orangutans, chimpanzees, and gorillas. It has been shown that while MHC class I first acquired its capacity to present antigen to T-cells, KIR began interacting with ligands formerly related to T-cell responses. Although T and NK cells have different roles, competing selection pressure on MHC class I genes resulted in MHC-C specialization upon NK responses. At the same time, MHC-A and MHC-B maintained their role in T-cell function (Augusto and Petzl-Erler, 2015). The orangutan is an evolutionary intermediary for the HLA-C1 epitope with its specific receptor KIR2. Both would have co-evolved. However, the origin of the HLA-C2 epitope needs to be clarified (Chazara et al., 2011).

Later in our evolutionary history, studies comparing Neanderthal and modern human sequences found that the HLA-C*07:02:01:01 allele probably comes from the cross between *H. sapiens* and Homo neanderthalensis and has been preserved to the present day in regions of Northwest and Southeast Asia. Furthermore, this allele has been linked with low susceptibility to autoimmune diseases such as diffuse cutaneous and systemic sclerosis and supports the hybridization between these two species theory y (Miren Ainhoa Riaño Vivanco, and Afonso, 2016). Finally, it is estimated that 1 million years ago, along with the brain development of hominids of modern human ancestors with allotypes (C1 and C2), a functional balance was reached with the KIR A and KIR B haplotypes, respectively, during gestation, preserving the immune functionality and reproductive purpose (Moffett and Colucci, 2015), the latter could be key for understanding the reason for the origin of this gene and the unique functional characteristics that have in *H. sapiens*.

Recent information suggests that HLA-C may have originated with another histocompatibility gene: HLA-G. These two genes are probably an adaptation in primate gestation because they actively participate in pregnancy’s development, tolerance, and immune response, expressed by intervillous trophoblasts (Carter, 2011; Li et al., 2021). However, HLA-C has the particularity of acting as a fetal alloantigen and deep invasion of trophoblast towards the placenta, a unique quality among the members of the class I histocompatibility complex and distinctive of human and certain types of apes pregnancy (Carter, 2011; Carter et al., 2015; Li et al., 2021).

## 2 Function

HLA-C is the most recently evolved member among the genes involved in the major histocompatibility complex. Its primary function is to present peptides to cytotoxic T lymphocytes with which it has contact in the cell membrane, in addition to serving as a specific ligand for KIR, a family of genes that encode recognition proteins in the NK cell membrane, actively participating in its development and regulation (Anderson, 2018; Souza et al., 2020).

The major histocompatibility complex class I, C [HLA-C], encodes glycoproteins distributed in the membrane of cellular tissues, platelets and mature erythrocytes (Barzuna, 2003). This protein plays an essential role in the immune response against bacterial and viral pathogens and in cancer and the rejection or acceptance of organs and tissues, preeclampsia in pregnant women, and rheumatic and autoimmune diseases such as psoriasis or alopecia areata (Haida et al., 2013; Siegel et al., 2019). The communication mechanism mainly mediates all these pathologies between HLA-C and NK cells, specifically by KIR signalling that can inhibit or activate the function of this group of lymphocytes (Laperrousaz et al., 2012).

The allele HLA-C*06:02 executes an autoimmune response against ADAMTS-like protein five presented to CD8^+^ T-cells as a causative melanocyte autoantigen; together, the allele, the autoantigen, and the T cell receptor (TCR) trigger a psoriasis autoimmune response (Arakawa et al., 2021). HLA-C*6 and its role in autoantigen response in epidermal interaction with CD8^+^ T-cells represent a risk of generating psoriasis (Mylonas and Conrad, 2018). In addition, LL37, an antimicrobial peptide (AMP) overexpressed in psoriasis skin that triggers activation of innate immune cells, shelters CD4^+^ and CD8^+^ T-cells (Lande et al., 2014).

The HLA-C gene is expressed in the maternal-fetal interface, playing an important role in immunomodulation for placentation and pregnancy wellbeing. In addition, due to high polymorphisms in KIR and HLA-C genes, the combination of fetal HLA-C and maternal KIRs will possibly determine a well-developed pregnancy (Souza et al., 2020).

As mentioned, this gene is expressed in intervillous trophoblasts of placental invasion in human pregnancy. This process occurs in several apes with these genes (Carter et al., 2015). It is interesting that in neonatal development, the interaction of HLA-C with KIR determines the risk of obstetric complications like small or large babies, this depending on the level of invasion trophoblasts that the alleles of HLA-C and KIR determinate: KIR AA + HLA-C2 = small babies, KIR2DS1 + HLA-C2 = large babies (Moffett et al., 2015).

## 3 Structure and expression

Structurally, HLA-C has eight exonic regions distributed along 3,388 bp, with 18.3% CpG islands and 66.1% Guanine-Cytosine, making HLA-C susceptible to epigenetics silencing phenomena such as methylation compared to the other members of the major histocompatibility complex, because although the number of CpG islands is very similar HLA-A (110 CpG), HLA-B (118 CpG) (HLA-C 116 CpG), HLAC concentrates the CpG islands in their promoter region. Exons 6, 7, and 8 have a relatively low level of CpG islands compared to the others that make up this gene, with exons 1, 2, and 3 being the densest in terms of guanine-cytosine. Remarkably, exons 2 and 3 polymorphisms give it the peptide binding specificity for the class I histocompatibility complex (Siegel et al., 2019). These characteristic polymorphisms of the HLA- (A, B, C) genes seem to be generated as an adaptive product to pathogen infection throughout our evolutionary history (Kulpa and Collins, 2011).

As a transcriptional product, HLA-C, like other members of HLA class I, generates a heavy polypeptide chain of 5 domains. The first two domains make a peptide-binding groove (α1, α2), and an additional one (α3) binds the remaining domains, transmembrane, and cytoplasmic tail. It is essential to mention that the α3 domain is non-covalently coupled to a beta 2-microglobulin (β2M) not encoded by the HLA complex but by a gene on chromosome 15 (Klein and Sato, 2000). Exons 2 and 3 encode α1 and α2 domains, respectively, and their variations directly impact HLA-C expression in the cell membrane. α3 is encoded by exon 4. Exon 5 encodes the transmembrane domain, exons 6 and 7 the cytoplasmic tail and exon eight corresponds to a polyadenylation region (poly A). Exon 1 transcribes the peptide targeting the cell membrane. However, it is not part of the functional structure of the HLA-C molecule (Souza et al., 2020).

**FIGURE 2:**
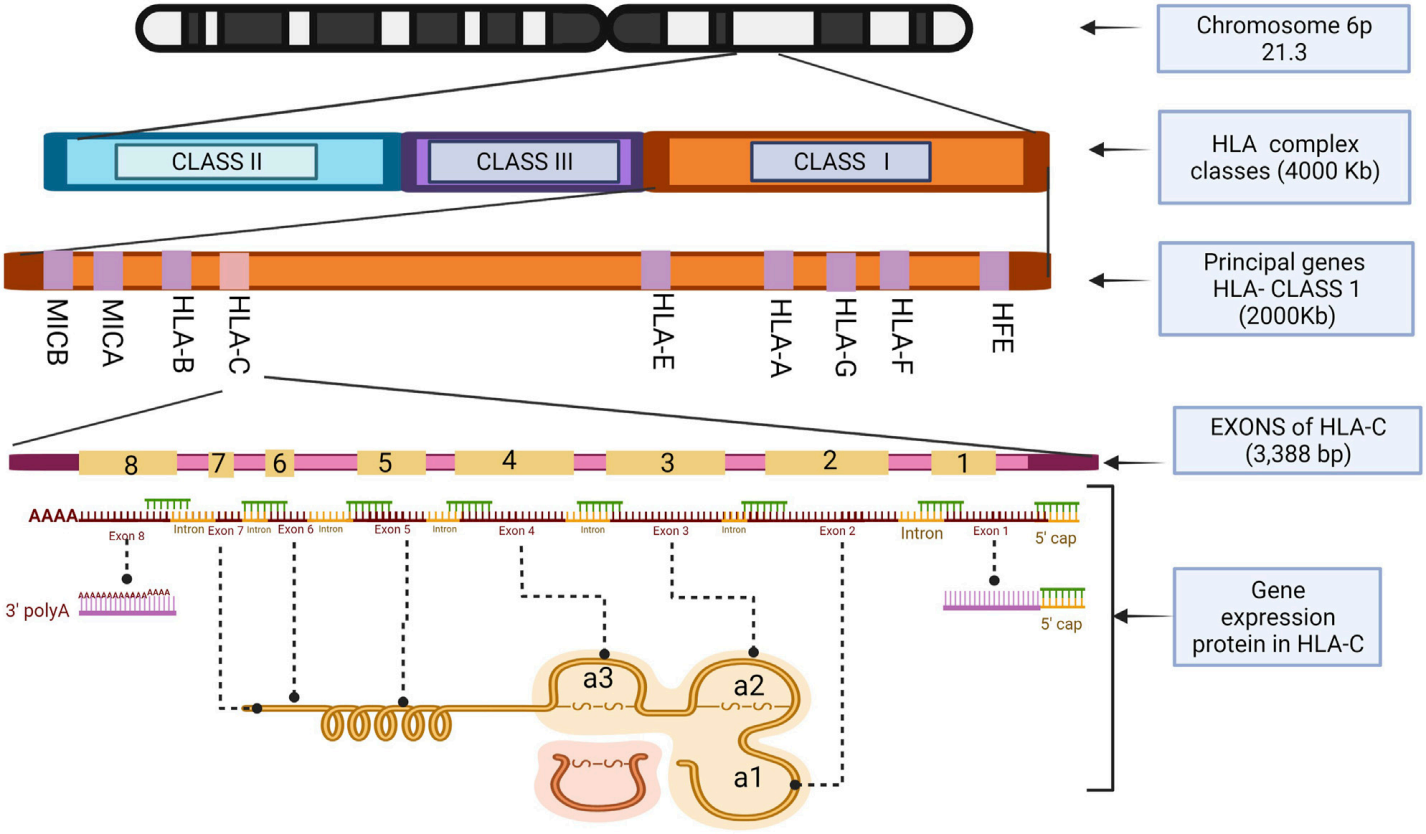
HLA-C exons location and the structure of the histocompatibility proteins. Illustrations Created with BioRender.com.

**FIGURE 3:**
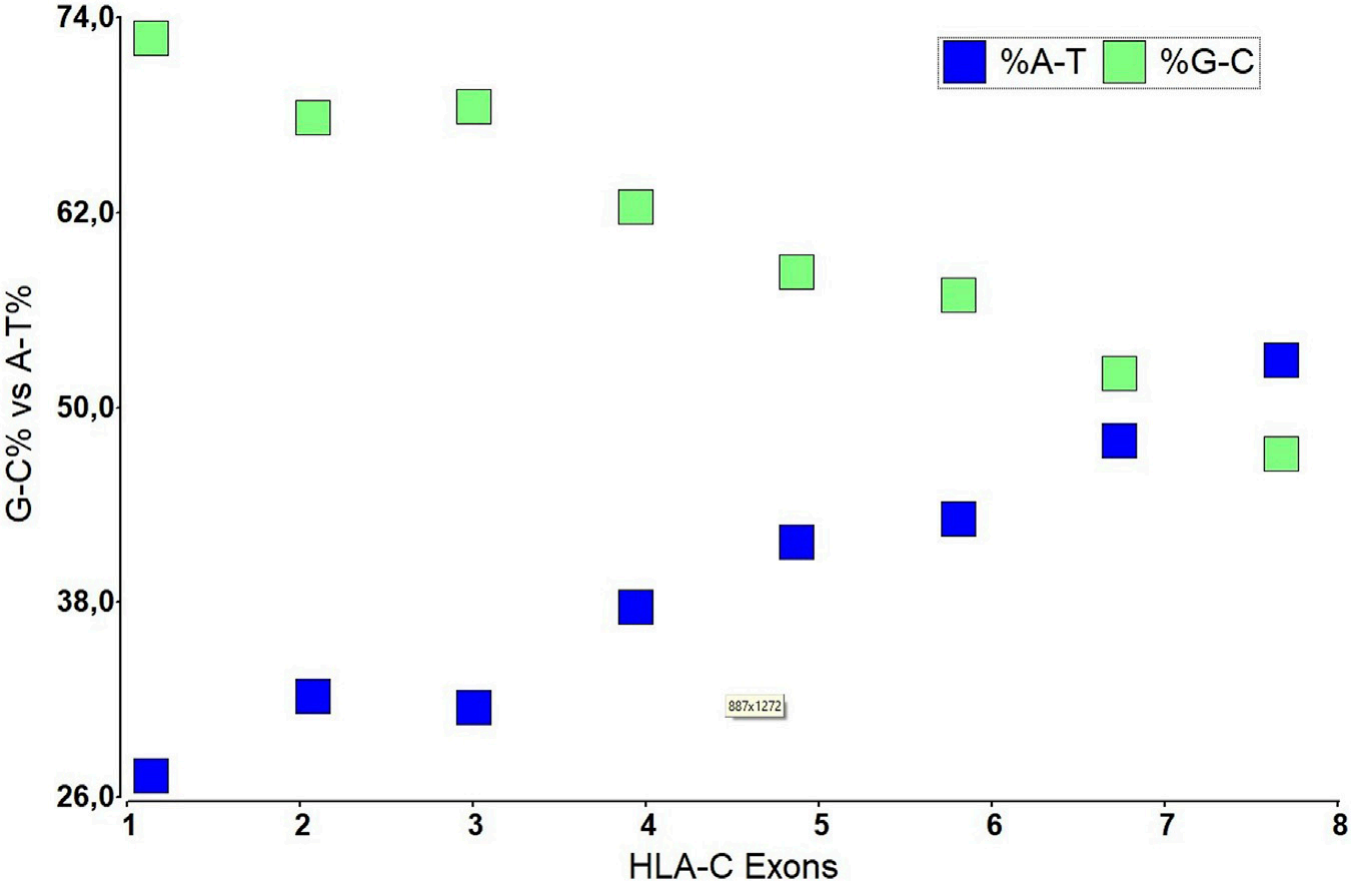
The nucleotide composition of HLA-C in each exon of its DNA structure.

The α1 helix domain of HLA-C is unusually conserved. The KYRV motifs (residues 66, 67, 69, and 76 in all alleles), in addition to a conserved glycine at amino acid 45 of pocket B, are unique characteristics of this gene, unlike the α2 domain, which is more like that of HLA-B (Blais et al., 2011). These small structural qualities decrease the presence of HLA-C in the cell membrane by reducing the amount of mRNA transcribed from it. There are also post-transcriptional control mechanisms that prevent the coupling of the protein on the cell surface (Zemmour and Parham, 1992; McCutcheon et al., 1995). It was recently identified that alternative splicing during the maturation of HLA-C mRNA regulates its expression depending on the polymorphisms present in exon 1 (Goodson-Gregg et al., 2019). The expression of HLA-C throughout the life of a human being is remarkably lower, about 13–18 times less than its paralogous genes HLA-A and HLA-B (Johnson et al., 2018). It has been determined that this lower expression is due to the heavy chains of HLA-C being poorly associated with microglobulin (β2M), which will form its functional structure in the cell membrane (Chazara et al., 2011). However, the expression control mechanisms for this gene have not yet been thoroughly investigated (Figures 2, 3).

## 4 Related pathologies

### 4.1 Infectious diseases

HLA-C is new in discovery and characterization if we compare it with its paralogous brothers (HLA-A and HLA-B), both in its role in the immune system and the particularities of its expression and structure (Kaur et al., 2017). In the early 1990s, its function in the human major histocompatibility complex was not known, only that it was the member of HLA class I with the lowest level of expression in cells (Snary et al., 1977; Dill et al., 1988). Today we know that HLA-C is highly relevant to the condition of the immune response. For example, a higher gene expression is associated with partial resistance to the HIV-1 virus: more particularly, a single nucleotide polymorphism (C/T) 35 bp upstream can cause the virus to proliferate the viral load in the organism of the carrier of this variant (Apps et al., 2013; Kaur et al., 2017). This polymorphism generates a relatively low protein expression on the cell surface as it is regulated by a microRNA that binds to these monopoly morphic regions, reducing the expression of HLA-C alleles. Despite this, microRNAs do not seem to be the only regulatory process nor the most effective for this gene (Kulkarni et al., 2011). Individuals with HLA-C*04:01 and HLA-C*12:03 haplotypes, in combination with certain types of HLA-A and HLA-B, could have partial protection or resistance to HIV by triggering an additive immune effect that better controls viral infection (Leslie et al., 2010).

In the case of genital herpes type II, it has been identified that the C*04 and C*02 alleles of HLA-C are present in individuals with greater susceptibility to infection and severe development of the pathology that could trigger neoplasms (Sasso et al., 2020).

Interestingly, for other viruses such as hepatitis C, HLA-C1 allotype confers resistance to Caucasian and African American individuals exposed to medium doses of the virus. However, at high doses, this “innate defence” is not enough to counteract the virus (Khakoo et al., 2004). These susceptibility or innate immunity mechanisms to viral infections are due to the interaction of HLA-C and its variants with KIR of NK cells and the not-so-specific interaction of HLA-C and its variants with cytotoxic T lymphocytes. It may also be due to the microRNA-mediated control mechanism of its expression and supposed coupling quality to the cell surface and viral proteins (Zipeto and Beretta, 2012).

HTLV-1 is a less well-known virus that affects T lymphocytes. Unfortunately, it has no cure and no successful treatments have yet been reported; in cases with a high viral load, it has been related as an etiological agent of leukaemia (Gotuzzo Herencia et al., 2010). A study in a Japanese population showed that the HLA-C*07:02 allele generates susceptibility to developing myelopathies associated with HTLV-1 while the C*08 allele protects the individual from suffering from these diseases even with the presence of the virus; despite this, the evidence suggests that there are other variants of HLA genes with an equal or more significant influence of this duality of susceptibility/resistance (Jeffery et al., 1999; Rafatpanah et al., 2007; Penova et al., 2021).

Epstein-Barr virus infection occurs during the first years of life. There is 5% of the worldwide population has a resistance mechanism to this virus that we do not understand yet. Studies of the US population found that individuals who appear to be immune to this virus have a variant of a single nucleotide (TT) at −35bp and express a lower amount of HLA-C protein (Durovic et al., 2013). It is not clear, but the Epstein-Barr virus is associated in some way with the development of multiple sclerosis, perhaps as a trigger when there is a predisposition, since a recent study found that KIR receptors, particularly in individuals with KIR2DS1, KIR2DS3, or KIR3DS1 alleles, present a more significant reactivation of the virus, all these receptors interact with HLA apparently (Wang et al., 2022).

Finally, due to its central role in activating NK cells, HLA-C has been linked mainly to viral infections, autoimmune diseases, and cancer. On the other hand, it has been proposed that more than a thousand bacterial species, including *Helicobacter, Chlamydia, Brucella*, and *Campylobacter,* trigger an immune response mediated by the HLA-C/KIR interaction (Sim et al., 2019).

### 4.2 Autoimmune diseases

As previously mentioned, HLA-C is susceptible to epigenetics silencing events or hyper-methylation, which could negatively condition its expression level. This process has been pointed out as an epigenetic marker for autoimmune diseases like psoriasis (Chen et al., 2016). Also, within the immunity spectrum, HLA-C has been linked to transplant rejection and skin diseases like alopecia areata or Crohn’s Disease, all related to an increased expression of this gene (Haida et al., 2013; Kulkarni et al., 2013; Khashaba, 2017).

In psoriasis vulgaris, the C*06:02 allele from HLA-C generates susceptibility to this chronic skin illness affecting around 2% of the world’s population. C*05 (along with other variants of HLA-A and B, C*07, and C*15 alleles) can promote disease protection or at least reduce susceptibility to this pathology. Environmental factors are additional risk factors which can further increase disease risk of pre-existing predisposition for the pathology (Cardili et al., 2016; Wiśniewski et al., 2018). Psoriatic arthritis is a common pathological condition in psoriasis patients. Some individuals have been identified with C*12, C*02, and C*06 alleles associated with such disorders as more severe psoriasis (Sokolik et al., 2014). Also, it has been determined that a decrease in carriers of HLA-C type receptors KIR2DS2 and KIR2DS1 reduces T and NK cell activation, enhancing this type of Arthritis (Martin et al., 2002). Another variation of Arthritis, without a relationship to psoriatic pathologies, is generalized osteoarthritis, shown in Japanese population research to be related to an HLA-C*04 allele found in many individuals suffering from this condition. On the contrary, C*1 and C*10 alleles were less common in this group of patients (Wakitani et al., 2001).

A similar case occurs in “Graves” disease, a pathology in which the immune system affects the thyroid. It has been found that individuals with HLA-C alleles such as C*03 and C*16 are unlikely to develop this pathology, while those with the C*07 allele are more susceptible to suffering from it (Simmonds et al., 2007). Even so, if an individual with the C*03:02 allele suffers from Graves’ disease, the usual treatment with methimazole can generate hepatotoxicity (Li et al., 2019a). Remarkably, the same gene causes a pathology that simultaneously determines liver disease susceptibility and treatment response. Of special note, the liver can also be affected by autoimmune hepatitis modulated by the allotype C*07:01 of HLA-C (Strettell et al., 1997).

Alopecia areata is a disease caused by genetic and epigenetic immune irregularities, but the presence of C*04:01 and C*15:02 alleles of HLA-C predisposes this pathology in the Japanese population (Haida et al., 2013). In a Brazilian population study, individuals with no familial history of alopecia areata but suffering from different degrees of this pathology were analyzed. They showed a higher frequency of the C*04 allele, but no other significant differences were found between the control groups (Barbosa et al., 2016). However, more recent studies that analyzed the top class I histocompatibility genes propose that HLA-A and B alleles represent a more significant risk factor than HLA-C alleles. The C*07:02 allele was more frequent in individuals with alopecia areata. However, its mechanism still needs to be fully understood. Still, stress conditions are supposed to promote an inflammatory HLA-B and C response which over-activates a variety of T lymphocytes, affecting a specific area of the scalp. This mechanism is similar in individuals holding the C*15 allele, found in people with capillary density loss both in the beard and eyebrows (Hayran et al., 2021).

Other immune disorders in which HLA-C participates are systemic lupus erythematosus and progressive systemic scleroderma, suggesting that these pathologies could be linked to variants of KIR receptors. Accordingly, it was found that patients with these diseases are carriers of the KIR2DS1 and KIR2DS2 alleles, both HLA-C receptors which promote different severity levels of these diseases (Pellett et al., 2007). In the case of lupus, it was found that individuals with the HLA-C1 allele or carriers of HLA-C*17:01 are susceptible to this disease, apparently due to a greater expression of the HLAC1/KIR2DS2 ligand, resulting in an increased cytoplasmic antioxidant response due to oxidative stress in patients with systemic lupus erythematosus (Gambino et al., 2018; Hanscombe et al., 2018).

Crohn’s disease or syndrome is related to an immune imbalance that affects the mucosa of the intestinal tract. A study conducted in the Korean population found that an intergenic region between HLA-C and HLA-B called: rs114985235 and the HLA-C*01 allele is associated with developing this pathology (Jung et al., 2016). Still, unlike the previously mentioned pathologies, this immune disorder does not depend on the HLA-C/KIR interaction.

Another disease, primary sclerosing cholangitis, is caused by an autoimmune disorder that generates a progressive and degenerative inflammation of the liver and bile ducts (Vargas, 2018). In a study of the European population, it was found that the HLA-C1 allele is associated with a greater tendency to suffer from this disorder. In contrast, the HLA-C2 allele significantly reduces the risk of primary sclerosing cholangitis (Hov et al., 2010). In chronic cases of this disease, malignant carcinoma can develop, resulting in the need for liver transplantation treatment. A study linked to liver transplant rejection found that the HLA-C*07 allele generates a greater risk for acute rejection mediated by infiltration of NK and T-cells in the transplanted organs (Fosby et al., 2014).

A study of the Japanese population found that the HLA-C*12:02 allele protects against a muscle disorder known as idiopathic inflammatory myopathy, in which HLA-C and other members of the major histocompatibility complex class I are highly expressed in the affected necrotic myofibers (Seki et al., 2019).

Finally, those variants in HLA-C expression have been linked during fetal development with preeclampsia, obstructed delivery, and even with a low weight of the neonate (Moffett and Colucci, 2015).

**FIGURE 4:**
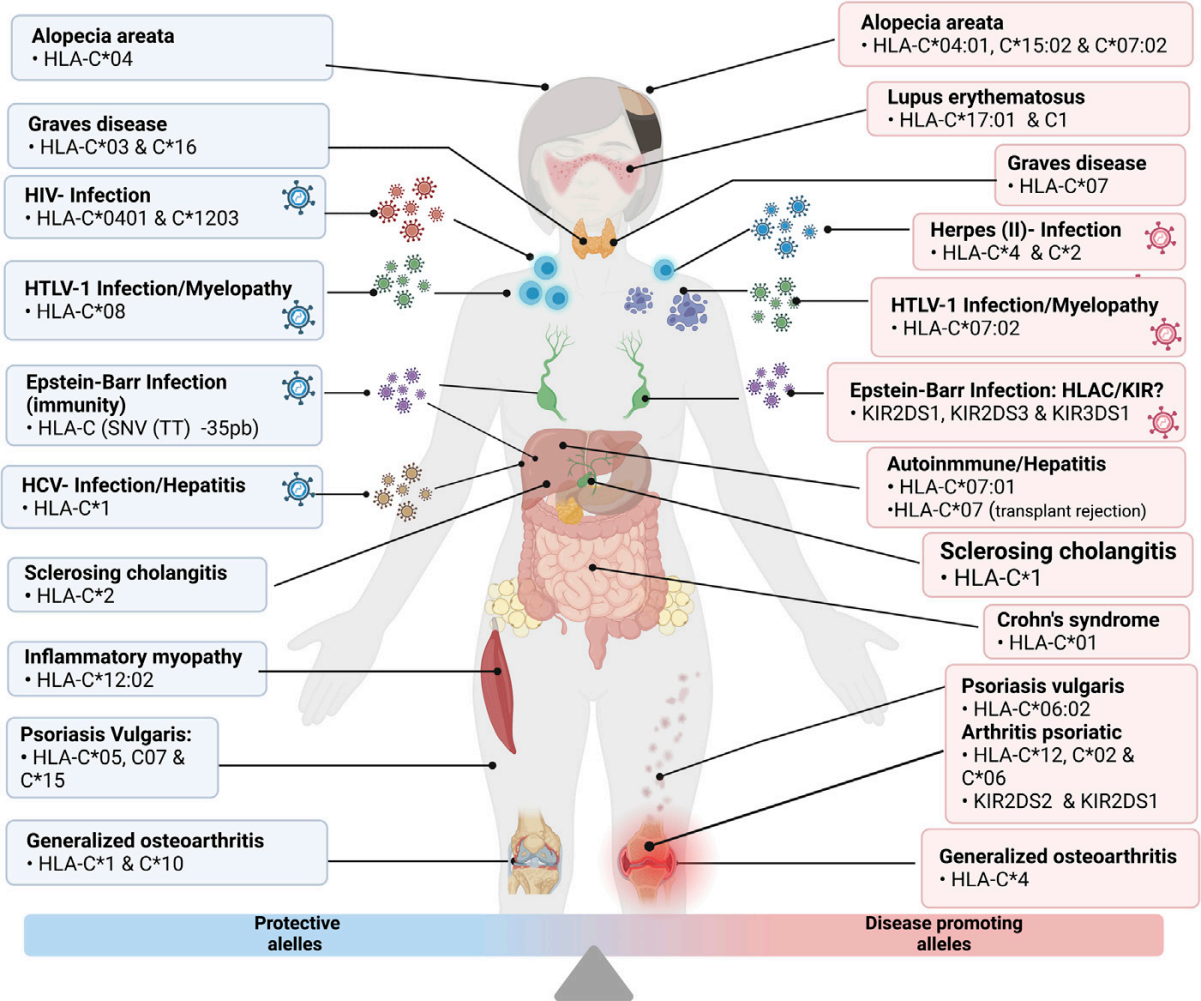
Grouping of the disease and protective alleles caused by the different variants of HLA-C described in detail in the previous points “Infectious Diseases” and “Autoimmune Diseases”. Illustrations Created with Bio Render.com.

All the diseases mentioned above are graphically detailed in Figure 4.

### 4.3 Cancer

The immune response is highly linked to cancer pathologies, where chronic inflammation in the tumour microenvironment represents a key hallmark of cancer. Reciprocally, a deficiency of the immune system also increases susceptibility to tumorigenesis, whereas the recognition of cancer cells is modulated by the histocompatibility complex of immune cells (Sondka et al., 2018). A decrease in the expression of HLA class I have been linked to tumour progression, metastasis, and reduced survival. However, the exact level of expression of these genes in different types of cancer is still unclear, and in some cases, the evidence is contradictory (Powell et al., 2012). It is also necessary to mention that deficiencies in the histocompatibility recognition system are not the only deficiency by which cancer cells escape immune control, as senescent cells can generate cancer dormancy-resistance during chronic cancer treatment, and clonal variation of cancerous cells can emerge without being identified as harmful by the organism defences (Fouad and Aanei, 2017).

It is currently considered that structural alterations in the HLA complex trigger an evasion of the immune system in cancerous tissue because the β2M macroglobulin, which is part of the final protein structure of histocompatibility class I, is considered a tumour suppressor gene in addition to being catalogued as a cancer hallmark which promotes cancer escape from immune surveillance (Castro et al., 2019; Tate et al., 2019).

HLA-C’s characteristic immune activity of interacting with natural killer cells (NK) was discovered at the beginning of the century. Consequently, it was proposed as a highly relevant protein for oncological studies (Falk and Schendel, 1997). A few years later, this hypothesis was further strengthened since structural allelic variants and expression of this gene were linked to aggressive neoplasms in nasopharyngeal and cervical cancer, both caused by viruses, raising the possibility that the interaction of HLA-C/KIR as an innate immune response is involved in activity and viability of cancerous cells (Butsch Kovacic et al., 2005; Martin et al., 2010).

HLA/KIR signalling activates NK cells that induce natural cytotoxicity through membrane receptors characteristic of this group of cells (Biassoni et al., 2001). This mechanism eliminates potentially harmful cells, highlighting the HLA-C/KIR2DS1 interaction in which their different alleles determine the level of tolerance or cellular cytotoxicity; this is very relevant in cancer typologies such as acute myeloid leukaemia in which the success of stem cell treatments depends on the histocompatibility of the allografts and the HLA-C/KIR2DS1 typology in donor and HLA-C1 in recipient generates a low relapse rate by inducing an anti-leukemic effect, while an individual homozygous of HLA-C2 poses a considerable risk (Venstrom et al., 2012).

A Caucasian population study from Poland found that the homozygous HLA-C1 epitope in the presence of KIR2DL2 and KIR2DS2 receptors caused lung cancer patients to have more than twice the life expectancy of individuals with other histocompatibility alleles (Wiśniewski et al., 2012). There is a paradoxical role for HLA-C in oncological pathologies, as evidence shows both low and high-expression gene correlations with various types of cancer. For example, the HLA-C*04 and HLA-C*15 alleles have been linked to a greater tendency to develop papillary thyroid carcinoma. In contrast, the HLA-C*07:01 allele protects against this pathology, at least in the Chinese population (Shuxian et al., 2014). In contrast, in the Saudi population in Arabia, no difference was found between patients and control groups when analyzing the HLA-C1 and -C2 alleles in a colorectal cancer study (Al Omar et al., 2015).

Studying the alleles and structural variants of HLA-C seems very relevant to understanding the aggressiveness of different types of cancer. However, the variability of this gene between populations could represent a challenge. For example, in the Chinese people, it was found that the allele HLA-C*08:01 is a risk factor for developing adenocarcinoma (Li et al., 2019b). In clinical cases, it has been reported that the loss of expression of an allotype very similar to HLA-C*08:02 generates an immune evasion in tumour tissue (Tran et al., 2016). Recently it was found that a decrease in the frequency of HLA-C*08:01 and an increase in HLA-C*04:01 is associated in the Korean population with the risk of generating glioblastoma (Choi et al., 2021). Also, in this population, it was recently found that overexpression of HLA-C decreases the cell viability of colorectal cancer, exerting a strong influence on distinct cancer pathways such as JAK/STAT, retinoblastoma and Hedgehog signalling (Lim et al., 2022). Also, overexpression of the HLA-C gene has previously been associated with low survival expectancy of cancer or the development of autoimmune diseases.

The immune relevance in cancer is closely related to HLA-C. For example, it has been shown that there is a significant decrease in immune activity in colorectal cancer due to a reduced expression of HLA-C in the tumor that did not present genetic structural alterations but rather epigenetic modifications. That reduced its expression level, allowing cancer to proliferate (Kawazu et al., 2022). This is corroborated by other recent investigations, which suggest that HLA-C demethylation levels and HLA class 1 could be epigenetic markers of prostate cancer (Rodems et al., 2022).

Despite the above, cancer as a pathology is developed by escaping a malignant cell from the immune system. If this system is not functioning competently, it facilitates these aberrant cells to proliferate and generate tumours, at least in the case of solid tumours. The methylation of the HLA class can cause this deficient expression I complex, as reported in gastric cancer (Ye et al., 2010).

## 5 After SARS-COV2 outbreak

During the 2020 outbreak caused by the severe acute respiratory syndrome coronavirus 2 (SARS-CoV2), polymorphisms in HLA-C were correlated with a higher mortality rate due to viral infection by triggering an overactive immune response SWED4Wlinked to the KIR mechanism of NK cells (Wang et al., 2020a; Sakuraba et al., 2020; Khor et al., 2021). Polymorphisms HLA-C*14:02, HLA-C*07:29, HLA-C*08:01 in the Chinese population, HLA-C*12:02:02:01 in the Japanese population, HLA- C*17 in the Italian population, HLA-C*04:01 in German, Spanish, Swiss, and American populations, HLA-C* 16 and HLA- C*01 in the Spanish population and occasionally HLA-C*04:01:01:01 in India were all significantly associated with SARS-CoV2 disease severity.

## 6 Discussion

All the examples and comparisons cited seem to indicate significant involvement of HLA-C regulation in different types of cancer and infectious or autoimmune pathologies, where the structural variations (alleles) of the gene represent both potential risk and possible protection against some dysfunction of the organism, mediated by the mechanism of HLA-C and KIR. Epigenetic features and polymorphic characteristics represent parallel evolutionary strategies to balance health advantages and disadvantages for survival benefits. Further studies are needed to compare HLA allele frequencies from different populations to estimate the relative disease risk for each HLA-C allotype.

The physiological involvement mechanisms of HLA-C are far from being fully understood, and new pathological association mechanisms continue to be discovered. For example, this year, a commonly used antibiotic “trimethoprim-sulfamethoxazole” was found to trigger an extremely rare and lethal type of respiratory failure that appears to be generated exclusively in individuals carrying the C*07:02 allotype and its paralogue HLA-B*07:02 (Goldman et al., 2022). Another example is Graves’ disease, where individuals with HLA-C*03:02 allele were more susceptible to acquiring a methimazole-induced liver injury (Li et al., 2019a). This indicates the need to expand the spectrum from an understanding of HLA-C involvement in pathologies (alleles of susceptibility or resistance) towards how the structural variability of this gene determines susceptibility or not for a high spectrum of pathologies, as mentioned in this article.

In a study of malignant neoplasms, the importance of the KIR-HLA-C variants was highlighted when applying chemotherapy treatments and drugs such as “rituximab”. It was shown that those HLA-C2 homozygotes did not benefit from the drug, while those with the KIR2DS1-HLA-C variant considerably increased their survival rate (Kaddu-Mulindwa et al., 2022).

In conclusion, finding new allelic variants in different populations around the world of the major histocompatibility complex class I type C (HLA-C) has become commonplace due to the novel relevance of this protein for the prevention, diagnosis, prognosis, and correct treatment of a wide variety of pathologies that cover a large part of the human health spectrum. Therefore, future studies focusing on better understanding HLA-C gene variant associations with treatment will need personalized pharmaco (epi) genomic applications in different demographic groups.
